# Prostate Club-like Cells Reveal Context-Dependent Epithelial States in Homeostasis Remodeling and Cancer

**DOI:** 10.3390/cells15131133

**Published:** 2026-06-23

**Authors:** Shuai Tang, Ximo Wang, Kian Fogarty, Fangmin Chen, Kai Li, Minghao Zhang, Mingui Fu, Benyi Li

**Affiliations:** 1 Department of Urology, Central Hospital, Tianjin University, Tianjin 300170, China; tangshuai@tju.edu.cn (S.T.); wangximo@126.com (X.W.); mrlikai211@163.com (K.L.); zmh_0504@126.com (M.Z.); 2 College of Medicine, Nankai University, Tianjin 300071, China; 3 Department of Urology, The University of Kansas Medical Center, Kansas City, KS 66160, USA; kfogarty2@kumc.edu; 4 Department of Urology, Wuhan Sixth Hospital, Wuhan 430015, China; 5020201287@nankai.edu.cn; 5 Department of Biomedical Science, School of Medicine, University of Missouri Kansas City, Kansas City, MO 64108, USA; fum@umkc.edu

**Keywords:** prostate club-like cells, epithelial heterogeneity, noncanonical epithelial states, prostate cancer, benign prostatic hyperplasia

## Abstract

**Highlights:**

**What are the main findings?**

- Prostate club-like cells are best understood as context-dependent noncanonical epithelial states rather than as a fully established lineage.
- Club-like programs recur in the prostatic urethra and proximal ducts and reappear in benign remodeling, inflammatory lesions, prostate cancer, and treatment-associated adaptation.

**What are the implications of the main findings?**

- This framework helps clarify prostate epithelial heterogeneity, spatial specialization, and state transitions across homeostatic and disease contexts.
- Harmonized nomenclature, longitudinal sampling, spatial validation, and functional perturbation are needed to define the biological meaning of prostate club-like states.

**Abstract:**

Prostate club-like cells have emerged as a recurrent but conceptually unsettled epithelial population across normal prostate, benign remodeling, inflammatory lesions, and prostate cancer. Although the term derives from airway biology, current evidence suggests that, in the prostate, these cells are better viewed as context-dependent noncanonical epithelial states than as a definitive lineage. Single-cell, spatial transcriptomic, and integrative studies place club-like cells most consistently in the prostatic urethra and proximal ducts under near-homeostatic conditions, whereas related programs reappear in benign prostatic hyperplasia, proliferative inflammatory atrophy, and tumor-associated niches. Across these contexts, club-like states intersect with androgen perturbation, inflammatory remodeling, epithelial plasticity, and treatment adaptation. Molecularly, they are defined less by a single marker than by a partially overlapping secretory, stress-associated, and remodeling-related gene program, with variable relationships to urethral luminal, intermediate, and progenitor-like epithelial states. This review synthesizes current evidence on the definition, distribution, molecular identity, functional implications, and disease relevance of prostate club-like cells. We argue that their main significance lies in clarifying prostate epithelial heterogeneity and state transitions, while key priorities include harmonized nomenclature, longitudinal sampling, spatial validation, and functional perturbation.

## 1. Introduction

Club cells were first described in the bronchiolar epithelium of the small airways and are now recognized as non-ciliated secretory epithelial cells involved in host defense, xenobiotic metabolism, and epithelial repair after injury [1]. In the prostate, however, the term club-like is used by analogy rather than by established lineage equivalence. High-resolution profiling studies have shown that the prostate epithelium extends beyond the classical basal, secretory luminal, and neuroendocrine framework and includes additional epithelial states distinguished by anatomical location, regenerative behavior, and transcriptional identity [2,3]. Single-cell mapping of normal human prostate and prostatic urethra identified SCGB1A1-associated club cells and KRT13-associated hillock cells in the urethral and proximal ductal compartment, whereas other studies identified anatomically distinct luminal progenitor populations at distal invagination tips and demonstrated broader regenerative potential across luminal cells than older hierarchical models had assumed [4,5]. These observations provide the context for discussing prostate club-like cells: not as a settled lineage, but as part of a broader landscape of epithelial heterogeneity and noncanonical luminal identity.

This distinction matters because the prostate and lung are not equivalent epithelial systems. What carries over from the airway literature is the concept of a secretory, stress-responsive epithelial program, not proof of a shared developmental origin between prostate club-like cells and airway club cells [1,2]. In normal prostate tissue, club-like populations are most consistently localized to the prostatic urethra and proximal ducts rather than to the conventional acinar luminal compartment [2,3]. That proximal compartment has also been linked to urethral luminal epithelia that appear relatively castration insensitive, further complicating their relationship to previously proposed facultative progenitor-like states [3]. Accordingly, this review uses prostate club-like cells as a working epithelial concept rather than as a definitive lineage label. In practical terms, “club-like” refers here to epithelial cells or states showing a recurrent secretory and stress-associated luminal module, often including SCGB-family genes, PIGR, MMP7, CP, LTF, and related ductal or luminal epithelial features.

The issue becomes more consequential in disease contexts. In benign prostatic hyperplasia (BPH), 5α-reductase inhibitor (5ARI) exposure has been associated with a shift from conventional luminal features toward a more club-like transcriptional state [6]. Spatially resolved analyses of nodular BPH have likewise highlighted remodeling-associated epithelial changes and noncanonical epithelial subpopulations in hyperplastic tissue [7]. In proliferative inflammatory atrophy, club-like cells have been identified in inflammation-associated peripheral-zone lesions and were reported to overlap with intermediate luminal states [8]. In localized prostate cancer, single-cell analysis has identified tumor-associated club-like epithelial states, and more recent spatial transcriptomic work has linked related programs to reduced androgen signaling, luminal progenitor-like features, and immunosuppressive myeloid niches in a subset of tumors [9,10]. Cross-dataset integration further suggests that periurethral-like, ductal-like, and other noncanonical epithelial programs recur across regression, regeneration, and cancer rather than remaining confined to a single static compartment [11]. Taken together, these studies suggest not that every club-like state is biologically identical, but that related epithelial programs repeatedly emerge in settings of androgen perturbation, inflammation, benign remodeling, and neoplasia.

The current evidence is therefore both persuasive and incomplete. It is persuasive because club-like programs have now been captured across normal tissue, BPH, PIA, and prostate cancer using single-cell, spatial, and integrative approaches [2,3,6,12]. It is incomplete because most of these data define transcriptional states and anatomical associations, whereas direct lineage tracing in the human prostate is not feasible [4,5,7]. In this review, prostate club-like cells are treated as a recurrent noncanonical epithelial program whose importance lies in linking epithelial heterogeneity, regional specialization, tissue remodeling, and disease relevance. The key questions are how these cells should be defined, how they relate to other noncanonical luminal states, where they are found, and how strongly current evidence supports a meaningful role for them in prostate biology and disease.

## 2. Defining Prostate Club-like Cells: Concept, Terminology, and Boundaries

The term club-like cells should be used as a descriptive label rather than as a definitively established cell identity. Club cells were originally defined in the small-airway epithelium as non-ciliated secretory cells, and the prostate field adopted this label because subsets of prostate epithelial cells showed partial transcriptional similarity to that airway program, not because cross-organ lineage equivalence had been demonstrated [1,2]. In the normal human prostate atlas, SCGB1A1-associated epithelial cells were annotated as club cells, whereas a separate KRT13-associated population was annotated as hillock cells [2]. This terminology was helpful because it drew attention to epithelial diversity in the prostatic urethra and proximal ducts. At the same time, it imported a naming system that can sound more lineage-definitive than the evidence warrants. For that reason, club-like is best understood here as a prostate-centered descriptor for a recurring epithelial program, not as proof that these cells are the direct prostate counterpart of airway club cells [1,2].

A second challenge is that club-like sits within a broader and only partly harmonized nomenclature. The original atlas separated club and hillock populations within the prostatic urethra and collecting ducts [2]. Subsequent work on urethral luminal epithelia reframed much of this territory in more explicitly prostate-centered anatomical terms and argued that several markers previously interpreted as facultative progenitor markers, including TACSTD2/TROP2, LY6A/Sca-1, KRT4, and PSCA, were, in fact, labeling proximal urethral luminal cells [3]. More recent cross-dataset meta-analyses have further suggested that cells previously labeled Club and Hillock map onto broader luminal ductal (LumDuctal) and prostatic urethral (PrU) populations and that the one-to-one correspondence between older labels and stable biological entities is weaker than the early nomenclature implied [11]. This shift does not invalidate the earlier terminology, but it does mean that club-like, hillock-like, urethral luminal, LumDuctal, and periurethral-like labels cannot be treated as either synonymous or fully discrete. A more accurate view is that they describe overlapping regions of a proximal noncanonical epithelial landscape whose exact boundaries vary by dataset, sampling context, and annotation strategy [3,11]. In this review, “club-like cells” is used when the evidence refers to an anatomically or spatially identifiable epithelial population, whereas “club-like program” or “club-like state” is used when the evidence primarily reflects a recurrent transcriptional pattern detected across different contexts. For future studies, a practical approach is to use anatomical or compartment-level terms such as prostatic urethral, PrU, or LumDuctal when the primary evidence reflects spatial localization or cross-dataset compartment mapping and to use club-like or hillock-like terminology for marker-defined states or subprograms within this broader proximal noncanonical epithelial territory. Cluster annotations should therefore specify the marker module, anatomical or disease context, and comparator population rather than relying on a single historical label.

The boundary between club-like cells and intermediate or progenitor-like epithelial states is equally important. Distal luminal progenitors have been identified at the tips of prostate invaginations, indicating that not all noncanonical luminal states occupy the same anatomical niche [5]. Regeneration studies have also shown that luminal plasticity is more broadly distributed across surviving luminal cells than would be expected if epithelial recovery depended on a single rare subtype [12]. More recently, work on Janus kinase/signal transducer and activator of transcription (JAK/STAT)-dependent intermediate Basal-B cells has emphasized that the prostate contains intermediate epithelial states with their own regulatory logic and that these states cannot simply be collapsed into either a club-like or a mature luminal category [13]. This distinction becomes even clearer in transformation models. In an ETS-related gene (ERG)-driven initiation model, highly proliferative intermediate cells were shown to coexpress basal, luminal, hillock, and club marker genes, indicating that club-associated signatures can emerge as part of a broader mixed epithelial program rather than as an isolated identity [14]. Accordingly, club-like should not be used as a blanket term for every epithelial state that differs from the conventional acinar luminal phenotype. Although some club-like states overlap with intermediate or progenitor-like programs, this overlap is only partial. A related issue is the meaning of club-like shifts across biological contexts. In non-neoplastic tissue, the term most often refers to a proximal epithelial population enriched in the urethra and collecting ducts [2,3]. In BPH, however, 5α-reductase inhibitor exposure has been associated with a transition from conventional luminal features toward a more club-like transcriptional program, suggesting that club-like identity can also emerge through epithelial remodeling rather than through simple persistence of a pre-existing compartment [6]. In proliferative inflammatory atrophy, club-like cells were identified in inflammation-associated peripheral-zone lesions and were closely linked to intermediate luminal states [8]. In localized prostate cancer, tumor-associated club-cell states remained recognizable, but their molecular profile no longer matched normal proximal club cells and instead overlapped with altered androgen-response and progenitor-like features [10]. In advanced disease, this contextual drift appears even more pronounced: double-negative castration-resistant prostate cancer (CRPC) has been reported to express a mixed basal, club, and hillock epithelial identity rather than a cleanly separable club-cell program [15]. These observations make it difficult to defend a single essentialist definition broad enough to encompass normal proximal club cells, BPH-associated luminal-to-club transitions, PIA-associated intermediate lesions, and tumor-associated mixed epithelial states as though they were all the same biological object.

Consistent with this practical definition, prostate club-like cells are used here to denote epithelial populations that recurrently display a partially overlapping secretory and stress-associated luminal module, while club-like programs or states refer to the transcriptional pattern itself. This definition is narrower than all noncanonical luminal cells but broader than SCGB1A1-positive urethral cells alone. The module often includes SCGB-family genes together with PIGR, MMP7, CP, LTF, and related ductal or luminal features, and is most often encountered in proximal urethral, ductal, inflammatory, hyperplastic, or tumor-remodeled contexts [6,10]. Two constraints remain important. First, club-like cells are not equated with all urethral luminal or PrU populations, because those broader compartments include cells without a clear club-like signature [3,11]. Second, club-like cells are not equated with all intermediate or progenitor-like states, because prostate epithelial plasticity extends beyond the club-like category itself [5,14]. Keeping those constraints in place is essential; otherwise, the label becomes so broad that it loses interpretive value. A recent review of gland- and cell-level heterogeneity in the prostate reaches a similar practical conclusion: club and hillock populations are biologically informative, but their value lies in illustrating epithelial diversity and transition states rather than in proving a finalized lineage map [16].

## 3. Evidence Base and Methodological Framework

Current knowledge of prostate club-like cells is derived primarily from cell-state profiling rather than direct lineage demonstration. The field therefore rests first on descriptive evidence: single-cell atlases of normal human and mouse prostate identified noncanonical epithelial populations in the urethral and proximal ductal compartment, including club- and hillock-associated states, and later studies detected related epithelial programs in benign hyperplasia, PIA, and localized prostate cancer [2,3,4]. These datasets established that club-like programs recur across multiple biological settings, but they did not by themselves determine whether all of these states represent one conserved epithelial entity or several context-dependent variants [7,8,10].

A second layer of evidence comes from anatomical validation. Here, transcriptomic clustering has typically been paired with immunostaining, RNA in situ hybridization, or spatial transcriptomics to determine where club-like programs are located in tissue. This combination has anchored club- and hillock-related populations to the prostatic urethra and proximal ducts in normal tissue, linked related programs to nodular remodeling in BPH, and identified club-like epithelial states in both inflammatory lesions and tumor-associated spatial niches [9,10]. These approaches are essential because cell-state annotation without anatomical context can easily blur urethral luminal, ductal, intermediate, and tumor-remodeled epithelial identities. At the same time, spatial co-localization remains correlational. It can show that a club-like program occupies a specific niche or repeatedly appears near particular stromal or immune populations, but it cannot by itself establish developmental ancestry or a stable cell-fate hierarchy [3,9,10].

Functional inference has been supported mainly by ex vivo culture systems. Organoid and primary-culture studies have shown that human prostate epithelial populations can preserve substantial heterogeneity outside the native gland and that luminal progenitor-like cells can generate prostate organoids under defined conditions [17]. These models are valuable because they allow perturbation, clonogenic assessment, and closer examination of epithelial plasticity. They also have clear interpretive limits. Single-cell analysis of primary human prostate cells in monolayer and organoid culture showed that intermediate epithelial states can be preferentially expanded ex vivo, which means that organoid output should not be treated as a neutral mirror of in vivo epithelial composition [17]. In addition, tissue dissociation and cell-recovery procedures may differentially preserve or deplete fragile, ductal, club-like, or intermediate epithelial states before culture is even established. For club-like cells specifically, this is important because secretory-stress, progenitor-like, and intermediate programs may partially overlap in culture, even when they are more anatomically constrained in tissue [18].

The strongest mechanistic evidence still comes from mouse lineage and perturbation models. Lineage-tracing studies have shown that adult basal and luminal compartments are largely self-sustained during homeostasis [19]. Regeneration studies demonstrated that luminal recovery after androgen withdrawal and restoration can be driven by a broad range of persisting luminal cells rather than by a single rare stem-like subset [12]. Inflammatory injury can reopen basal-to-luminal differentiation routes, and developmental single-cell analyses have shown that epithelial fate is shaped by stromal androgen signaling and broader cell–cell communication networks [20,21]. More recent multi-omic integration studies have further refined state definition by linking transcriptional heterogeneity to chromatin-level regulatory architecture in prostate cancer samples [22]. Taken together, these approaches define the current evidentiary boundary of the field. They strongly support the existence of recurrent noncanonical epithelial programs, including club-like states, but they still fall short of proving that club-like populations described across normal prostate, BPH, inflammatory lesions, and cancer occupy a single fixed lineage position.

## 4. Club-like Cells Within Prostate Epithelial Hierarchy

Prostate epithelial hierarchy is still most commonly described in terms of basal, luminal, and neuroendocrine compartments. In adult tissue, lineage-tracing studies support the view that basal and luminal populations are largely self-sustained during homeostasis [19]. Developmental studies complicate this picture, however, because both multipotent and unipotent progenitors contribute to postnatal prostate growth [23]. The hierarchical position of club-like cells therefore cannot be inferred from adult homeostatic lineage behavior alone; it must be considered in relation to regional epithelial specialization and the broader plasticity of the gland.

Current evidence places club-like cells on the luminal side of the epithelial hierarchy, but outside the conventional acinar luminal compartment. In the normal human prostate, SCGB1A1-associated club cells and KRT13-associated hillock cells are localized predominantly to the prostatic urethra and proximal ducts rather than to distal secretory acini [2]. Urethral luminal epithelia in this proximal compartment are relatively castration insensitive, which further distinguishes them from the dominant androgen-dependent luminal population [3]. Cross-species single-cell analyses have also identified conserved proximal epithelial states with mixed or noncanonical luminal features [4]. Club-like cells are therefore best viewed as part of a proximal, duct-associated, noncanonical luminal domain rather than as a universal intermediate state between basal and luminal cells.

This does not mean that club-like cells are equivalent to all luminal progenitor or intermediate epithelial states. Distal luminal progenitors have been identified at the tips of prostate invaginations, indicating that at least one progenitor-like luminal population occupies a niche distinct from the proximal urethral territory in which club-like cells are usually found [5]. Luminal recovery after androgen restoration can arise from a broad population of surviving luminal cells rather than from a single rare stem-like subset [12]. Castration-resistant Nkx3.1-expressing luminal cells represent another rare stem-like luminal population with tumor-initiating potential [24]. Club-like cells therefore fit more naturally within a heterogeneous noncanonical luminal compartment than within a singular progenitor category.

The same issue becomes apparent when epithelial-state plasticity is considered. Urethral luminal epithelia persist after castration, supporting the existence of a durable proximal epithelial population with club-like features [3]. At the same time, cross-dataset integration across regression, regeneration, and cancer has shown that periurethral-like and ductal-like programs can recur in multiple biological settings [11]. JAK/STAT signaling maintains an intermediate epithelial population during prostate basal cell fate determination, indicating that mixed-state epithelial identities are part of normal prostate biology rather than a phenomenon confined to tumors [13]. In an ERG-driven initiation model, proliferative intermediate cells coexpressed basal, luminal, hillock, and club marker genes [14]. Considering these data, a rigid one-cell-type/one-position model is difficult to sustain.

A more plausible interpretation is a provisional two-layer framework of club-like identity. One layer reflects a proximal epithelial population with recognizable anatomical localization, whereas the other reflects a recurrent transcriptional program detected in intermediate, regenerative, or tumor-remodeled states. However, these two layers cannot yet be empirically separated with confidence using current cross-sectional single-cell or spatial datasets. Without longitudinal sampling, lineage tracing, or direct perturbation, it remains difficult to distinguish persistence of a proximal compartment, de novo state induction, transient state conversion, selective depletion, or annotation-dependent similarity across datasets. These layers are therefore conceptually useful, but they should not be collapsed into a single fixed hierarchical or lineage-based claim.

In this review, club-like cells are therefore placed provisionally within a noncanonical luminal compartment enriched in the proximal prostate, while related club-like programs in disease are interpreted as context-dependent transcriptional states rather than as evidence of continuous lineage progression. In this framework, prostate club-like cells are best viewed as part of a proximal noncanonical luminal landscape rather than as a fixed intermediate step in a linear epithelial hierarchy.

## 5. Anatomical and Pathological Distribution of Prostate Club-like Cells

The anatomical distribution of club-like cells is one of the strongest reasons not to treat them as a uniformly dispersed epithelial lineage. In the normal human prostate, SCGB1A1-associated club cells and KRT13-associated hillock cells were localized to the prostatic urethra and proximal collecting ducts rather than to the distal acinar compartment [2]. Urethral luminal epithelia, defined in later work, occupied the same proximal territory and extended into the proximal prostate, reinforcing the view that club-like identity is spatially linked to the urethral–ductal axis of the gland [3]. This proximal bias matters because it places club-like cells near the compartment most closely associated with periurethral remodeling rather than within the canonical secretory luminal compartment typically used to describe the peripheral acinar prostate.

This distribution becomes particularly relevant in BPH. Urethral luminal epithelia were reported to increase in glandular BPH nodules, and later work showed that 5α-reductase inhibitor exposure was associated with a transition from conventional luminal features toward a more club-like transcriptional state [6]. Spatial and integrative studies of BPH have also shown that nodular formation is accompanied by epithelial remodeling rather than by simple expansion of a single pre-existing epithelial class [7,25]. In practical terms, club-like programs in BPH may reflect both enrichment of a proximal epithelial compartment and inducible adoption of a club-like state during androgen-perturbed hyperplastic growth. This distinction is important because it avoids reducing BPH to a “club-cell disease,” while still recognizing that club-like programs are especially prominent in the periurethral and transition-zone setting in which BPH develops.

The picture changes in proliferative inflammatory atrophy. PIA has long been discussed as an atrophic but proliferative lesion associated with chronic inflammation and possible epithelial remodeling in the peripheral zone [26]. In this context, Huang and colleagues identified club-like cells in inflammation-associated PIA lesions and showed that club-cell gene expression in the peripheral zone was restricted to luminal epithelial cells classified as intermediate cells [8]. The important point is not simply that club-like signatures can be detected outside the urethral–proximal compartment. Rather, inflammatory stress appears capable of recruiting a club-like program into a lesion type that occupies a different anatomical zone and has historically been discussed within a carcinogenesis framework. The presence of club-like cells in PIA therefore broadens the spatial meaning of club-like identity without erasing the distinction between normal proximal club cells and inflammation-associated peripheral-zone club-like states.

In prostate cancer, distribution becomes more heterogeneous and more clearly niche-dependent. Single-cell analysis of localized primary tumors identified tumor-associated club-cell states rather than a uniformly distributed club-like compartment [10]. Spatial transcriptomic analysis later showed that club-like cells can occupy tumor regions associated with immunosuppressive myeloid inflammation, reduced androgen signaling, and treatment-relevant epithelial plasticity [9]. These findings do not imply that club-like cells are present in all tumors or that they occupy one consistent anatomic position across all specimens. Instead, they suggest a patchy distribution shaped by local epithelial state, microenvironmental context, and disease setting. This interpretation is also consistent with work on aggressive cribriform and intraductal carcinoma, in which single-cell analysis highlighted marked spatial and epithelial heterogeneity associated with aggressive morphology rather than a single unified tumor epithelial program [27]. Once cancer develops, club-like distribution appears less like a stable zonal map and more like a context-sensitive epithelial niche.

**Figure 1:**
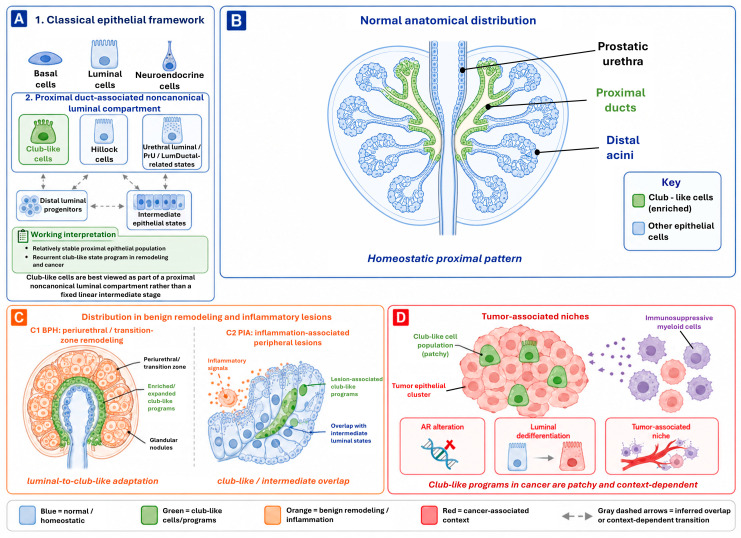
(**A**) Conceptual insert showing club-like cells within a proximal duct-associated noncanonical luminal compartment, with luminal-like intermediate cells positioned between canonical luminal and noncanonical luminal states. (**B**) In a normal prostate, club-like cells are enriched in the prostatic urethra and proximal ducts, consistent with a homeostatic proximal pattern. (**C**) In non-malignant pathological settings, club-like programs expand in periurethral/transition-zone remodeling in benign prostatic hyperplasia (BPH) and appear in inflammation-associated peripheral lesions in proliferative inflammatory atrophy (PIA), where they overlap with intermediate luminal states. (**D**) In prostate cancer, club-like programs are patchy and niche-dependent and are shown in relation to altered androgen receptor (AR) signaling, luminal dedifferentiation, and tumor-associated myeloid interactions.

Under near-normal conditions, club-like cells are concentrated around the urethral and proximal ductal compartment. In disease, related programs become detectable in periurethral hyperplasia, inflammation-associated peripheral lesions, and selected tumor niches. For the purposes of this review, the most useful way to conceptualize distribution is to distinguish a homeostatic proximal pattern from a pathology-expanded pattern. Figure 1 summarizes this anatomical and pathological distributional framework across normal prostate, benign remodeling, inflammatory lesions, and tumor-associated niches, with a small conceptual inset indicating the placement of club-like cells within prostate epithelial heterogeneity.

## 6. Molecular Identity and Candidate Regulatory Programs

The molecular identity of prostate club-like cells is best understood as a recurring marker module rather than as a single-marker checklist. In normal prostate and prostatic urethra, club-associated epithelial states were originally defined by SCGB-family expression together with secretory and duct-associated markers, whereas hillock cells were distinguished by a different KRT13-centered program [2]. In later prostate cancer datasets, the club-like signature remained recognizable but was no longer identical to the normal proximal pattern. Tumor-associated club-cell states were enriched for markers such as PSCA, PIGR, MMP7, SCGB1A1, and LTF [10]. A recent large-scale preprint atlas by Song et al. further identified an LTF/LCN2-high club-like tumor-cell population, supporting the view that tumor-associated club-like states may use a partially overlapping but context-shifted marker module [28]. In proliferative inflammatory atrophy, RNA in situ validation identified CP, LTF, MMP7, PIGR, and SCGB1A1 in lesion-associated club-like cells [8]. Across these settings, the most stable feature is not a single invariant gene but a partially overlapping secretory and stress-associated module that shifts with anatomical and pathological context. For that reason, a modular rather than essentialist definition is more useful at the molecular level.

This marker module also appears to combine several biological themes rather than pointing to a single lineage property. One component is clearly secretory, reflected by SCGB-family genes, PIGR, LTF, and CP [2,8,10]. Another is linked to proximal or duct-associated epithelial identity, which helps explain why club-like states were first recognized in the urethral–proximal compartment rather than in distal secretory acini [2,10]. A third is more closely related to epithelial stress, innate defense, or injury-associated remodeling, which is particularly evident in PIA and in tumor-associated club-like states [8,9]. Importantly, these themes do not remain identically coupled in every setting. In localized prostate cancer, one club-cell subtype retained club markers while also expressing luminal genes such as KLK2, KLK3, ACPP, and NKX3-1 [10]. This finding argues against a rigid separation between club-like and luminal programs and suggests that the molecular identity of club-like cells can include partial luminal re-entry rather than only luminal departure.

Androgen-response biology illustrates why the molecular identity of club-like cells should not be reduced to a single rule. Song et al. reported that LTF/LCN2-high club-like tumor cells showed enrichment of androgen-response, protein-secretion, and oxidative-stress pathways compared with benign club cells [10,28]. By contrast, Kiviaho et al. found that tissue areas enriched for club-like cells showed depleted androgen signaling, increased luminal progenitor markers, and a senescence-associated secretory phenotype [9]. Multi-omic analysis adds a third layer: Bian et al. identified a stemness-associated subset of club cells marked by SOX9-high and androgen receptor (AR)-low expression that became enriched after neoadjuvant androgen-deprivation treatment [22]. These findings are easier to reconcile if androgen signaling is viewed as a context- and comparator-dependent axis within club-like biology rather than as a fixed defining property. Benign or non-malignant club-like regions may appear relatively AR-low when compared with canonical luminal epithelium, whereas localized club-like tumor cells may show stronger androgen-response features when compared with benign club cells. The recurrent pattern is therefore not absolute AR loss but context-dependent reweighting of the luminal androgen-response program.

Candidate regulatory programs show a similar pattern of partial convergence rather than a single settled mechanism. Inflammatory and epithelial-stress signaling recur across multiple contexts, especially in PIA and in prostate cancer regions enriched for club-like cells [8,9]. JAK/STAT signaling is also relevant here, not because it has been established as a club-specific master regulator, but because it maintains an intermediate epithelial population during prostate basal cell fate determination and therefore helps define the broader mixed-state territory in which club-like programs can emerge [13]. In an ERG-driven initiation model, highly proliferative intermediate cells coexpressed basal, luminal, hillock, and club marker genes and were enriched for MYC, NF-κB, STAT, growth-factor, and stemness-associated programs while showing reduced AR-target expression relative to luminal cells [14]. These findings do not justify labeling JAK/STAT, NF-κB, or MYC as lineage-defining regulators of prostate club-like cells. They do, however, support a working framework in which club-like molecular identity is favored by inflammatory signaling, partial luminal reprogramming, and epithelial-state instability under regenerative or oncogenic stress.

**Figure 2:**
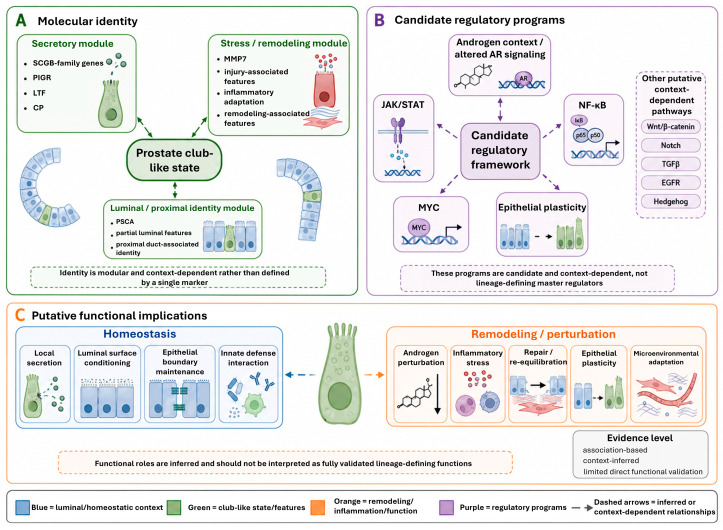
(**A**) Molecular identity of prostate club-like cells is depicted as modular and context-dependent, integrating secretory, stress/remodeling, and luminal/proximal identity features rather than being defined by a single marker. (**B**) Candidate regulatory links associated with prostate club-like states are summarized, including androgen context/altered AR signaling, JAK/STAT, NF-κB, MYC, and epithelial plasticity. (**C**) Putative functional implications of prostate club-like cells are shown across two broad contexts: homeostasis and remodeling/perturbation. In homeostasis, club-like programs are linked to local secretion, luminal surface conditioning, epithelial boundary maintenance, and innate defense interaction. In remodeling or perturbed states, they are associated with androgen perturbation, inflammatory stress, repair/re-equilibration, epithelial plasticity, and microenvironmental adaptation. Functional interpretations are intended as association-based and context-inferred, with limited direct functional validation.

The remaining question is whether club-like molecular identity represents one coherent regulatory state or a family of related states that converge on a similar marker module. Broader single-cell multi-omic studies suggest the latter is more likely. Integrated single-cell analysis of castration-resistant prostate luminal cells showed that noncanonical luminal states can be underpinned by distinct epigenetic architectures rather than by simple transcriptional noise [29]. Comparative transcriptomic analysis of double-negative prostate cancer likewise identified a mixed basal, club, and hillock epithelial identity, pushing the club-like signature even further away from the idea of a neatly isolated molecular class [15]. For the purposes of this review, the most workable interpretation is that prostate club-like cells are defined by a recurrent secretory-stress and remodeling-associated module whose composition is reshaped by location, androgen context, inflammation, and tumor evolution. Taken together, these observations support a modular and context-dependent view of prostate club-like molecular identity. Figure 2 integrates the marker modules and candidate regulatory programs discussed here and provides a conceptual bridge to the putative functional implications considered in the next section.

## 7. Putative Functional Implications in Homeostasis and Epithelial Remodeling

The functional significance of prostate club-like cells remains more inferential than experimentally settled. Much of the intuition surrounding this cell state comes from the airway, where club cells are non-ciliated secretory epithelial cells involved in host defense, xenobiotic metabolism, and epithelial repair after injury [1,30]. Prostate literature does not justify transferring those functions wholesale across organs, but the analogy remains informative. In normal prostate and prostatic urethra, club-like cells occupy a proximal epithelial compartment and express a secretory and stress-associated program rather than a purely canonical acinar luminal profile [2,17,22]. That combination makes it reasonable to discuss club-like cells as a candidate epithelial interface state adapted to local secretion, environmental exposure, and tissue maintenance, while recognizing that direct functional validation in prostate tissue is still limited.

In near-homeostatic tissue, the most plausible functions are local and epithelial rather than lineage-defining. SCGB-family genes, PIGR, LTF, CP, and related components of the club-like module are consistent with a secretory epithelial phenotype and have been observed in inflammation-associated PIA lesions [8]. Their enrichment along the urethral–proximal axis also fits a setting in which epithelial cells are positioned closer to urinary flow, periurethral inflammation, and ductal junctions than conventional distal acinar luminal cells [3]. Club-like identity may therefore reflect a regional specialization that helps maintain epithelial integrity at a boundary zone of the gland. This interpretation is stronger than any claim that club-like cells have already been proven to constitute a dedicated progenitor or repair compartment in the normal prostate.

Their functional relevance becomes easier to conceptualize when the prostate is perturbed. In BPH, 5α-reductase inhibitor exposure has been associated with a transition from conventional secretory luminal features toward a more club-like transcriptional state [6]. Spatial and single-cell studies of nodular BPH have likewise shown that hyperplastic growth is accompanied by epithelial remodeling rather than by simple amplification of one stable epithelial class [7,25]. A more recent finasteride-focused spatial analysis further supported treatment-associated epithelial redistribution in hyperplastic tissue [31]. These findings are consistent with a model in which club-like programs mark an epithelial state associated with reduced androgenic support and chronic periurethral remodeling. In this sense, the club-like state is biologically informative not because it has been shown to drive BPH, but because it appears to identify a luminal program detected under conditions that destabilize the conventional secretory phenotype.

The same logic extends to tissue repair and epithelial plasticity. Club-like cells are often discussed alongside progenitor-like or intermediate states because those categories converge most strongly during regeneration and remodeling. Cross-dataset analysis across regression, regeneration, and cancer has shown that periurethral-like and ductal-like epithelial programs can recur across biologically distinct settings rather than remaining confined to one limited compartment [11]. Primary prostate culture and organoid studies add an important caution here: intermediate epithelial states can expand ex vivo disproportionately, indicating that plasticity-associated programs may be selectively favored outside native tissue architecture [17]. Airway biology again offers a parallel rather than proof. In the lung, distinct stem-like populations can be found among club cells and are mobilized during regeneration [30]. The prostate evidence is not yet strong enough to claim an equivalent regenerative hierarchy, but it supports the more cautious view that club-like programs may be associated with epithelial-state changes during repair-related remodeling, reprogramming, or luminal-state adjustment.

Inflammation provides the clearest context in which these functional inferences converge. In proliferative inflammatory atrophy, club-like cells were identified within inflammation-associated peripheral-zone lesions and overlapped with intermediate luminal states [8,32]. In localized prostate cancer, spatial transcriptomics linked club-like epithelial regions to immunosuppressive myeloid neighborhoods, reduced androgen signaling, luminal progenitor-like features, and a senescence-associated secretory phenotype [9]. Apostolov et al. further identified MHC II-expressing club clusters in hormone therapy-naïve localized prostate cancer, suggesting antigen-presentation-associated epithelial features and a possible epithelial–immune interface for discrete club-like states [33]. Earlier work on CD38-low inflammation-associated luminal cells also showed that inflammatory epithelial states in the human prostate can display enhanced clonogenicity and tumor-initiating potential, although those cells should not be equated directly with club-like populations [34]. Taken together, these studies support a cautious association-based interpretation: club-like programs are repeatedly observed in epithelial states associated with inflammatory stress, altered androgen signaling, and microenvironmental remodeling.

## 8. Club-like Cells and Programs in Prostate Disease

The disease relevance of prostate club-like cells and related programs is not uniform across benign hyperplasia, inflammatory lesions, and prostate cancer. What recurs across these settings is not one fixed cell identity with a specific function, but a noncanonical epithelial program that becomes more visible when glandular architecture, androgen dependence, or epithelial–microenvironment relationships are being reworked. For that reason, disease relevance is discussed here in a context-specific and primarily association-based manner rather than as a single mechanistic narrative.

Benign prostatic hyperplasia is the clearest setting in which club-like programs expand in a nonmalignant disease context. The transition zone and periurethral region, where BPH develops, overlap anatomically with the proximal epithelial territory in which club-like and urethral luminal populations are most readily detected [3]. In human BPH tissue, 5α-reductase inhibitor exposure was associated with a luminal-to-club cell transition rather than simple preservation of a static luminal phenotype [6]. Spatial transcriptomic and single-cell studies of BPH further show that hyperplastic growth involves coordinated epithelial, stromal, and inflammatory remodeling rather than expansion of one stable epithelial class [25,35]. In the BPH single-cell atlas by Unno et al., epithelial cells were classified into basal, club, and luminal populations, with the club population enriched for PIGR, MMP7, LTF, and SCGB3A1, providing a direct BPH-specific reference point for club-like epithelial annotation [35]. A finasteride-focused spatial and single-cell analysis then demonstrated additional epithelial alterations after treatment, reinforcing the idea that epithelial-state redistribution is part of the hyperplastic response rather than an incidental by-product [31]. In this setting, club-like programs are most plausibly interpreted as markers of periurethral remodeling and androgen-perturbed luminal adaptation, not as a stand-alone pathogenic lineage that by itself explains nodular growth.

The interpretation shifts in proliferative inflammatory atrophy. PIA has long been discussed as an inflammation-associated lesion of the peripheral zone and has often been considered within broader models of epithelial injury and early carcinogenic remodeling [26]. Huang and colleagues identified club-like cells in PIA and showed that expression of club-cell–associated genes in the peripheral zone was primarily localized to a subset of luminal epithelial cells classified as intermediate cells [8]. A subsequent commentary underscored the same point from a disease-biology perspective: club-like cells in human prostate inflammation appear in luminal cells adjacent to inflammation in the peripheral zone, are commonly found in PIA lesions, and raise questions about function, tumorigenic capacity, and androgen dependence that remain unresolved [32]. This places PIA-associated club-like programs in a different disease frame from BPH. In BPH, the state remains closely tied to periurethral anatomy and androgen perturbation. In PIA, the same label points instead to inflammation-associated epithelial plasticity in a zonal context in which prostate cancer commonly arises. Current evidence supports association and biological plausibility here, but not a deterministic precursor sequence from club-like PIA to carcinoma.

In localized prostate cancer, the club-like program is best viewed as one epithelial state within a heterogeneous tumor ecosystem. Single-cell analysis of primary prostate cancer identified tumor-associated club-cell states rather than a uniformly distributed malignant compartment [10]. The same atlas by Song et al. also reported that LTF/LCN2-high club-like tumor cells were most prominent in localized disease and markedly reduced in advanced states, decreasing from 8.86% of epithelial cells in localized prostate cancer to 0.95% in CRPC prostate tissues and 0.15% in mCRPC [28]. Spatial transcriptomic work then linked club-like epithelial regions to immunosuppressive myeloid niches, reduced androgen signaling, luminal progenitor features, and a senescence-associated secretory phenotype [9]. Although not specific to club-like epithelial niches, recent clinicopathological evidence showing heterogeneous PD-L1 expression and its association with Gleason Grade Group, cribriform morphology, and shorter biochemical recurrence-free survival further supports the broader relevance of immune-biomarker heterogeneity in prostate cancer [36]. A later single-cell and spatial study of hormone-therapy-naïve localized prostate cancer described epithelial functional states and fibroblast phenotypes within localized tumors and reported expansion of club-cell phenotypes within the malignant spectrum, interpreting this pattern as part of luminal dedifferentiation rather than evidence of an isolated tumor lineage [33]. These studies make the disease argument more concrete. Club-like programs in localized cancer are not merely residual echoes of the normal proximal compartment; they are detected within tumor regions associated with epithelial plasticity and distinct stromal-immune contexts, although their direct contribution to disease behavior remains to be functionally defined. This localized, niche-associated pattern should be distinguished from therapy-adapted or advanced disease states, in which club-like signatures may have different abundance, molecular features, and biological implications.

Therapy-adapted and advanced disease states require a separate interpretation from localized primary tumors. In this context, Baurès and colleagues reported that pre-existing club-like cells can potentiate androgen-deprivation-therapy resistance in prostate cancer models and proposed early targeting of castration-tolerant club-like cells as a therapeutic strategy [37]. This finding should be interpreted alongside the atlas-level observation by Song et al. that LTF/LCN2-high club-like tumor cells are markedly reduced in CRPC prostate tissues and nearly absent in mCRPC [28]. These findings are not necessarily contradictory because they address different disease stages, model systems, treatment contexts, and cell-state definitions. Baurès et al. support a role for club-like or club-related epithelial states during early adaptation to androgen-deprived conditions in selected experimental contexts, whereas Song et al. suggest that LTF/LCN2-high club-like tumor cells do not persist as a quantitatively prominent compartment in established metastatic disease. A separate metastatic progression study also showed convergence of prostate cancer cells toward an inflammatory-like state during dissemination, and an integrated single-cell and spatial progression atlas linked lineage plasticity to immune-microenvironment remodeling across CRPC and neuroendocrine disease [38,39]. Taken together, these studies support a narrower conclusion: club-like programs may mark part of the epithelial state space associated with early therapy-adaptive tolerance or broader inflammatory-like plasticity in selected contexts, rather than serving as universal drivers of resistance or advanced progression.

**Figure 3:**
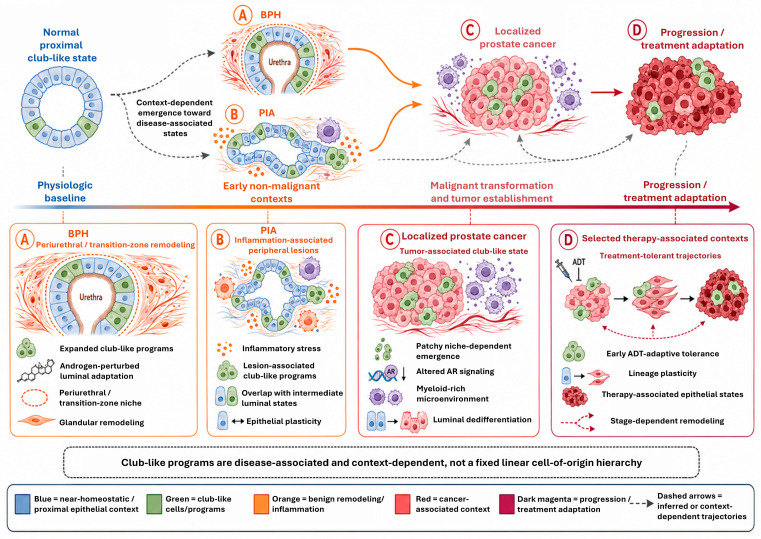
The upper trajectory presents a working model of context-dependent emergence and evolution of club-like programs from a normal proximal state toward progressively altered disease-associated contexts. (**A**) In benign prostatic hyperplasia (BPH), club-like programs are shown in periurethral/transition-zone remodeling and are associated with glandular remodeling and androgen-perturbed luminal adaptation. (**B**) In proliferative inflammatory atrophy (PIA), lesion-associated club-like programs are depicted within inflammation-associated peripheral lesions and are shown to overlap with intermediate luminal states in the setting of inflammatory stress and epithelial plasticity. (**C**) In localized prostate cancer, club-like programs are illustrated as patchy, niche-dependent epithelial states associated with altered AR signaling, a myeloid-rich microenvironment, and luminal dedifferentiation. (**D**) In therapy-associated contexts, club-like or club-related epithelial states are shown in relation to early ADT-adaptive tolerance, inflammatory-like plasticity, and stage-dependent remodeling, with LTF/LCN2-high club-like tumor cells becoming less prominent in CRPC and mCRPC.

The disease relevance of club-like cells and programs is therefore best understood in layers rather than as a single unified claim. In BPH, club-like programs are most closely tied to periurethral and transition-zone remodeling. In PIA, club-like cells appear within inflammation-associated intermediate epithelial states of the peripheral zone. In prostate cancer, club-like programs fit more convincingly within a broader noncanonical luminal repertoire associated with localized tumor heterogeneity, stromal-immune context, and, in selected settings, early therapy-adaptive tolerance. These observations support a context-dependent disease framework rather than a fixed causal hierarchy. Figure 3 summarizes the emergence of club-like cells and programs across benign remodeling, inflammatory lesions, localized prostate cancer, and selected therapy-associated contexts.

## 9. Current Limitations and Unresolved Questions

The main limitation of the current literature is not the absence of interesting epithelial states, but the difficulty of determining when similarly labeled states are in fact the same biological object. Across studies, the terms club, hillock, urethral luminal, PrU, LumDuctal, intermediate, and noncanonical luminal are often applied to populations that overlap only partially in marker composition, anatomical location, and inferred function [16]. Recent reviews of prostate gland- and cell-level heterogeneity have made the same point from a broader perspective: the field has moved rapidly toward finer epithelial classification, but terminology has not matured at the same pace [16]. This creates a practical problem for interpretation. A label such as club-like can be useful, but only if it is treated as a working descriptor rather than as proof that all similarly annotated populations are equivalent.

A second limitation is methodological. Single-cell and spatial studies have greatly improved anatomical resolution, and the recent adult human prostate spatial atlas underscores how heterogeneous the gland is even at baseline [40]. Even so, most available datasets remain cross-sectional, correlative, and dependent on tissue availability, dissociation strategy, and computational annotation. Direct lineage tracing in the human prostate is not feasible, and spatial co-localization alone cannot establish developmental ancestry or stable cell-fate relationships. This matters especially for club-like cells, because the field is currently more proficient at identifying where club-like programs appear rather than determining whether they arise from one persistent compartment, repeated state transitions, transient induction, selective loss, or annotation-dependent convergence across datasets.

A third unresolved question is whether normal proximal club-like cells, BPH-associated luminal-to-club transitions, PIA-associated club-like intermediate cells, tumor-associated club-like states, and therapy-tolerant club-like populations should be interpreted as one continuum or as several related but nonidentical phenomena. Existing data support both continuity and divergence. Cross-dataset meta-analysis has shown that periurethral-like epithelial programs can recur across regression, regeneration, and cancer [11]. At the same time, disease-focused studies increasingly suggest that club-like signatures are reshaped by inflammatory stress, androgen deprivation, stromal context, and tumor evolution [8,12,37,38]. Spatial multi-omics has added another layer of complexity by identifying club-like enrichment even in noncancerous glands adjacent to aggressive disease, raising the possibility that field effects and gland-level context influence how club-like states should be interpreted [41]. The label is therefore useful, but not yet precise enough to substitute for a context-specific definition.

What the field needs next is not simply more cluster names, but a more practical reporting framework for club-like states. As provisional criteria, future studies should specify whether “club-like” refers to a spatially identifiable epithelial population or to a transcriptional program, report the marker module used for annotation, and define the anatomical or disease context and comparator population. A minimal marker framework may include SCGB-family genes together with PIGR, MMP7, CP, LTF, and related ductal or luminal features, with LCN2 considered particularly relevant in tumor-associated LTF/LCN2-high club-like states. These markers should not be treated as a fixed checklist, but as a context-dependent module that requires spatial validation and cross-dataset comparison when possible. More broadly, key priorities include matched longitudinal sampling before and after treatment, tighter integration of single-cell and spatial data across disease stages, and analytically interpretable models that connect epithelial states to spatial niches and clinical outcomes [40,42,43]. Recent review literature has already noted that new single-cell-defined cell signatures in BPH and prostate cancer are accumulating faster than they are being biologically consolidated [42]. That imbalance is especially relevant here. Until club-like states can be tracked more rigorously across anatomy, disease stage, and therapeutic pressure, the central question will remain open: does the club-like label capture one prostate epithelial entity or a family of related epithelial solutions that recur under different constraints?

## 10. Conclusions

Prostate club-like cells are most convincingly understood as a recurrent noncanonical epithelial program rather than as a fully settled lineage. Across the current literature, related states have been identified in the normal urethral–proximal compartment, in benign hyperplasia, in proliferative inflammatory atrophy, and in localized and treatment-relevant prostate cancer settings. What persists across these contexts is not one fixed molecular or anatomical identity, but a recognizable combination of secretory, stress-associated, and remodeling-linked epithelial features that reappears when prostate epithelial organization is regionally specialized, perturbed, or reconfigured.

The main value of this framework is interpretive rather than taxonomic. It allows club-like states to be discussed in relation to epithelial heterogeneity, spatial context, inflammatory remodeling, and treatment adaptation without forcing them into a single definitive lineage model. It also clarifies why the same label can refer to somewhat different biological entities in normal tissue, BPH, PIA, localized cancer, and advanced disease. The central unresolved issue is therefore not whether club-like cells “exist,” but whether the current label captures one prostate epithelial entity or a family of related epithelial solutions that emerge under different constraints. That question will not be answered by additional cluster naming alone. It will require tighter nomenclature, more consistent cross-dataset mapping, longitudinal and treatment-aware sampling, and functional validation capable of distinguishing persistent compartments from recurrent state transitions.

## Data Availability

No new data were generated or analyzed in this study.

## Abbreviations

The following abbreviations are used in this manuscript:

5ARI	5α-reductase inhibitor
ADT	androgen deprivation therapy
AR	androgen receptor
BPH	benign prostatic hyperplasia
CRPC	castration-resistant prostate cancer
ERG	ETS-related gene
JAK/STAT	Janus kinase/signal transducer and activator of transcription
LumDuctal	luminal ductal
NF-κB	nuclear factor kappa B
PIA	proliferative inflammatory atrophy
PrU	prostatic urethral
